# Demonstration of tantalum as a structural material for MEMS thermal actuators

**DOI:** 10.1038/s41378-020-00232-z

**Published:** 2021-01-15

**Authors:** Longchang Ni, Ryan M. Pocratsky, Maarten P. de Boer

**Affiliations:** 1 CMU Mechanical Engineering Dept., 5000 Forbes Ave., Pittsburgh, PA 15213 USA; 2 Present Address: Fischione Instruments, 9003 Corporate Cir, Export, PA 15632 USA

**Keywords:** Structural properties, Electrical and electronic engineering, Structural properties

## Abstract

This work demonstrates the processing, modeling, and characterization of nanocrystalline refractory metal tantalum (Ta) as a new structural material for microelectromechanical system (MEMS) thermal actuators (TAs). Nanocrystalline Ta films have a coefficient of thermal expansion (CTE) and Young’s modulus comparable to bulk Ta but an approximately ten times greater yield strength. The mechanical properties and grain size remain stable after annealing at temperatures as high as 1000 °C. Ta has a high melting temperature (*T*_m_ = 3017 °C) and a low resistivity (*ρ* = 20 µΩ cm). Compared to TAs made from the dominant MEMS material, polycrystalline silicon (polysilicon, *T*_m_ = 1414 °C, *ρ* = 2000 µΩ cm), Ta TAs theoretically require less than half the power input for the same force and displacement, and their temperature change is half that of polysilicon. Ta TAs operate at a voltage 16 times lower than that of other TAs, making them compatible with complementary metal oxide semiconductors (CMOS). We select *α*-phase Ta and etch 2.5-μm-thick sputter-deposited films with a 1 μm width while maintaining a vertical sidewall profile to ensure in-plane movement of TA legs. This is 25 times thicker than the thickest reactive-ion-etched *α*-Ta reported in the technical literature. Residual stress sensitivities to sputter parameters and to hydrogen incorporation are investigated and controlled. Subsequently, a V-shaped TA is fabricated and tested in air. Both conventional actuation by Joule heating and passive self-actuation are as predicted by models.

## Introduction

Thermal actuation has proven to be a robust actuation technique in the microelectromechanical system (MEMS) field and has found many applications, including disk drives^1^, micro- and nanopositioners^2^, microgrippers^3^, scanning probes^4^, optical attenuators^5^, micromirrors^6^, gyroscopes^7^, linear and rotary microengines^8^, switches^9^, and nanomechanical property test platforms^10,11^. While traditional parallel plate or comb-driven electrostatic actuators require high driving voltages (≥30 V) and generate forces rarely exceeding 10 μN^12^, thermal actuators (TAs) use a lower operating voltage (5–10 V) yet provide a higher output force that is on the order of mN^13^. Compared with other common actuation mechanisms involving piezoelectric materials, magnetic materials or shape memory alloys, the use of which can be prevented by processing constraints, TAs are generally compatible with standard MEMS manufacturing methods, are easily scalable in size, and have a more compact structure^14^.

For MEMS TAs, polycrystalline silicon (polysilicon, PS) has been the dominant structural material^1,6,8,10–13^. However, PS TA legs typically operate at temperatures 300–500 °C higher than the substrate to generate useable displacement and force output because the coefficient of thermal expansion of PS (CTE, *α*_Si_ ≈ 2.7 µε/°C^15^) is the same as that of the silicon substrate. This causes relatively large power consumption, which is a well-known drawback^16^. In addition, structural PS is typically annealed at 1000 °C to control the residual stress^17^, a temperature that is incompatible with many processes and materials, thus prohibiting postprocessing on complementary metal oxide semiconductor (CMOS) foundry parts. The operating voltage of 5–10 V causes PS TAs to be at best indirectly compatible with CMOS, which typically operates at ≲1 V. New TA structural materials are desired that (a) have a high CTE, (b) can be deposited with low intrinsic residual stress and a low stress gradient, (c) can operate at lesser power and lower voltage, and (d) can be processed at lower temperatures than existing TAs.

We loosely place TAs in one of three categories: (i) hot-and-cold-arm (or U-shaped), (ii) chevron type (or V-shaped), and (iii) bimorph structures. These three types of TAs are actuated using different geometric form factors. Correspondingly, these are (i) asymmetric thermal expansion, (ii) constrained thermal expansion in one direction, and (iii) differences in the CTE values of two adjacent layers. Bimorphs employ at least one structural material other than PS. A low CTE layer is typically a dielectric or semiconductor material, while a high CTE layer is typically composed of a metal or polymer^14^. Because of this configuration, important compromises are made. For example, the motion results from a bending moment and is not rectilinear^12^. In addition, bimorphs typically produce out-of-plane motion due to the planar deposition of standard MEMS fabrication methods^14^. These actuators are fundamentally different from U-shaped and V-shaped TAs, which utilize homogeneous structural materials and do not rely on materials with different CTEs. Therefore, we limit our scope in this introduction to (i) U-shaped and (ii) V-shaped TAs using a single structural material.

Relatively few efforts to fabricate TAs using materials other than PS have been reported. The most common alternative is electroplated Ni^18,19^. Ni devices were fabricated in LIGA (German acronym that means lithography, electroplating, and molding, respectively) or in metal multi-user MEMS processes (metalMUMPs), which can allow for high-aspect-ratio structures. We note that the aspect ratio (thickness/width) is important because it determines whether the TA deflects in-plane or out-of-plane. Compared with TAs fabricated by conventional surface micromachining, these TAs are generally ≈10 times larger, and hence, the general benefits inherent in compact actuators, such as improved device density and overall system size reduction^16^, are lost. Other issues regarding Ni TAs, including mechanical stress-limited operation and undesirable out-of-plane buckling, have been reported^19^. Another widely studied structural material is the negative photoresist SU-8 (refs. ^20,21^). As a polymer, SU-8 has a large CTE (52 µε/°C). However, since it is not electrically conductive, a built-in metal heater is needed for actuation. Additionally, scaling down the size is difficult due to fabrication limitations^3^. Therefore, at a large thickness of tens of micrometers, SU-8 has mainly been reported to be used as a microgripper for biospecies manipulation due to its biocompatibility^21^.

Alternate TA structural materials fabricated by conventional surface micromachining that have been reported are Al and Au^22–24^. Pustan et al. reported the design and modeling of a switch made of Al based on V-shaped TAs^22,23^. However, limited details on microfabricated switches have been reported, and actuation has not been demonstrated. In any case, Al has its own limitations, including low strength and low melting temperature *T*_m_.

Mølhave et al. reported U-shaped microgrippers made of Au that was patterned by the lift-off technique^24^. Au was selected for demonstration purposes and because its thermal variations in piezoresistivity are smaller than those of silicon. The microgrippers had integrated deflection and force measurement capability that was implemented by measuring the differences in the piezoresistive changes in shortened and elongated beams. Limited by the resolution of the lift-off process, the devices had a low aspect ratio of 1:2 with a height *h* = 1 μm and a beam width *w* = 2 μm. The authors reported that the microgrippers failed to function properly due to out-of-plane bending. Indeed, the authors asserted that PS is a better structural material than Au with increased sensitivity because its gauge factor is ≈25 times greater than Au. Characterization of the structural material properties was not reported for Al or Au in these studies^22–24^.

Here, we demonstrate a refractory metal as a new structural material for micromachined TAs. The metal is fully compatible with a standard micromachining process flow. With its high *T*_m_ = 3017 °C, a refractory material possesses excellent thermal stability, and as a metal, its electrical conductivity is high, which in turn means its power consumption is <50% that of PS. However, many other criteria must be satisfied for the metal to be competitive with PS, which has long been the workhorse structural material in MEMS. Among these criteria, it should be possible to anisotropically etch layers with a ≳ 2:1 aspect ratio. The residual stress and residual stress gradient should be controlled to desirable levels. The temperature of an annealing step that controls stress should be as low as possible, certainly well below 1000 °C. The metal should be compatible with a sacrificial material, and its strength should be high. Although our main focus here is on demonstrating functioning TAs, we also demonstrate these above-mentioned attributes of tantalum (Ta).

Of the common refractory metals, Ta is the most desirable for TAs because it has a large CTE (*α*_*Ta*_ = 6.3 µεC) compared to Mo (*α*_*Mo*_ = 4.8 µεC) or W (*α*_w_ = 4.5 µε/°C)^25^. In principle, *isothermal* self-actuation is enabled due to the CTE mismatch between Ta and the Si substrate. This can be useful for temperature sensing^19^. A related application of particular interest to us is nanomechanical testing^10,11^, where in principle, stress can be applied merely by raising the temperature while the specimen remains isothermal.

In what follows, we model and compare the predicted TA behavior for Ta and PS (subsection “Model”). We then assess whether Ta can be integrated into the MEMS fabrication process (subsection “Preliminary experiments to assess whether Ta can be integrated into MEMS”). Issues that are addressed include material phase selection, strength and sensitivity to high-temperature annealing, CTE, residual stress control, and etchability. In subsection “Full fabrication sequence”, we integrate Ta into a full TA fabrication process. We find that the release etch causes a 1.2 GPa compressive stress change and show how this can be resolved. In subsection “Thermal actuator chartacterization”, we show that measured deflections match predictions both for Joule heating and for self-actuation.

## Results and discussion

### Model

**Fig. 1:**
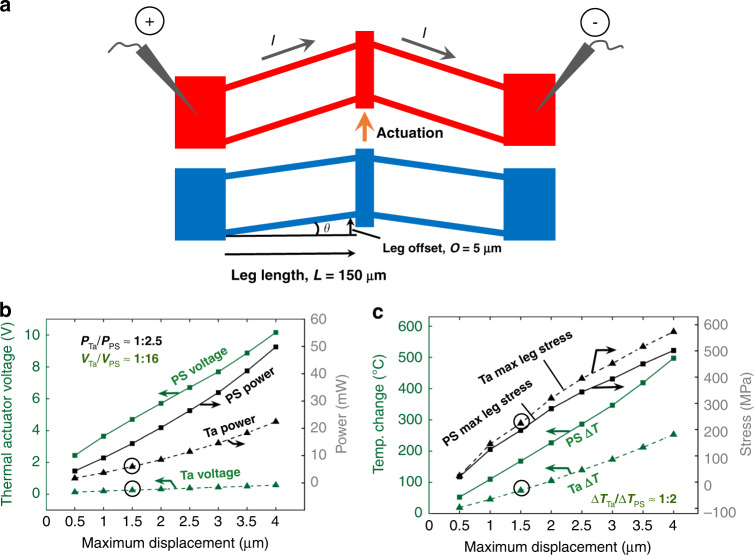
Modeled comparison of Ta between polysilicon (PS) thermal actuators. **a** Schematic of V-shaped thermal actuators. Dimensions indicated are assumed throughout this article, **b** comparison of actuation voltages and powers, and **c** maximum temperature and stress for PS and Ta actuators at different maximum displacements, as predicted by the electro-thermo-mechanical model. The circles indicate operating points discussed in subsection “Model”

V-shaped or chevron-type TAs are characterized by one or more inclined beams (or “legs”) arranged in parallel. They generate rectilinear deflection by thermal expansion. A schematic of a typical V-shaped TA is shown in Fig. 1a, and this TA is generally actuated by Joule heating. The modeled PS and Ta TAs have two leg pairs with a leg length *L* *=* 150 μm and an offset *O* = 5 μm, as defined in Fig. 1a. This same leg geometry is studied throughout this work.

To compare the actuation voltage and power consumption of Ta and PS TAs, we estimate the steady-state behavior with a simplified 1-D heat flow condition. This provides perspective in terms of the relative performance of the two materials. However, actuation by Joule heating is inherently a complex multiphysics problem. Therefore, we utilize a coupled electrothermomechanical model to predict TA behavior for further validation. For self-actuation, analytical solutions are readily available, and we develop a finite element analysis (FEA) model using ANSYS. These processes are described in detail, as follows.

#### Actuation by Joule heating

Let us assume a zeroth-order model in which the power *P* is the same for Ta and PS TA legs of the same cross-sectional area *A* and length *L*. Then

1 $$P = \frac{{V^2}}{R} = \frac{{V_{{\mathrm{Ta}}}^2}}{{\rho _{{\mathrm{Ta}}}\left( {L/A} \right)}} = \frac{{V_{{\mathrm{PS}}}^2}}{{\rho _{{\mathrm{PS}}}\left( {L/A} \right)}},$$

where *V* is the applied voltage, *R* is the electrical resistance, and *ρ* is the electrical resistivity. The subscripts Ta and PS denote the Ta and PS actuators, respectively. The (*L*/A) terms cancel. Measured data give *ρ*_Ta_ = 20 µΩ cm and *ρ*_PS_ = 2000 µΩ cm.

For a small gap between the legs and the substrate, the major source of heat loss in a gaseous atmosphere is thermal conduction from the legs to the substrate and depends only weakly on the TA material. Hence, we further approximate that for the same power, the increase in actuator leg temperature, Δ*T*, is the same. However, *α*_Ta_ ≈ 6 µε/°C, while *α*_PS_ ≈ 3 µε/°C. Therefore, for the same power delivered, the Ta TA deflects twice as much as the PS TA. If the Ta TA power *P* is halved, its deflection is the same as that of the PS TA. Hence, we take *P*_Ta_/*P*_PS_ = 0.5. With this consideration, Eq. (1) is rearranged, and values are inserted:

2 $$\frac{{V_{{\mathrm{Ta}}}}}{{V_{{\mathrm{PS}}}}} = \sqrt {\left( {\frac{{P_{{\mathrm{Ta}}}}}{{P_{{\mathrm{PS}}}}}} \right)\left( {\frac{{\rho _{{\mathrm{Ta}}}}}{{\rho _{{\mathrm{PS}}}}}} \right)} = \sqrt {\left( {\frac{{0.5}}{1}} \right)\left( {\frac{{20}}{{2000}}} \right)} \approx \frac{1}{{14}}.$$

Therefore, to zeroth-order, for the same deflection, a Ta TA operates at half the power and a 14 times lower voltage than the PS TA. As PS actuators typically operate at 5–10 V, Ta TAs operate at 0.5–1 V.

A coupled MATLAB/ANSYS simulation is used to model the thermal conduction paths in detail^13^. We use bulk Ta material properties from the refs. ^25–27^ for modeling. This model has been validated for PS, and we use it to refine the zeroth-order estimates.

In cross-section, the leg thickness *t* is 2.5 μm, while the width *w* is 1 μm. These choices are important and are explained as follows:

(i) A beam width of 1 μm is readily within our optical lithography capabilities. This ensures a well-controlled output force, which is highly sensitive to the change in beam width^13^.
(ii) The critical out-of-plane buckling stress, $\sigma _{\mathrm{c}}^{{\mathrm{op}}}$, must be higher than the critical in-plane buckling stress, $\sigma _{\mathrm{c}}^{{\mathrm{ip}}}$ to ensure the desired in-plane motion. We evaluate the worst-case scenario, i.e., a beam offset angle *θ* = 0, for two beams that are mirrored and connected at the shuttle. For unconstrained actuation, a sufficient condition is *t* > *w*. However, the ratio *t*:*w* must increase further if the in-plane motion of TA is constrained. For example, during nanomechanical tensile testing, TAs are used to apply a load, and their in-plane motion is constrained by the specimen. For constrained in-plane motion, the effective length of the legs doubles, and then

3 $$\frac{{\sigma _{\mathrm{c}}^{{\mathrm{op}}}}}{{\sigma _{\mathrm{c}}^{{\mathrm{ip}}}}} \approx \frac{{\pi ^2Et^2/\left( {3 \times \left( {2L} \right)^2} \right)}}{{\pi ^2Ew^2/\left( {3 \times \left( L \right)^2} \right)}} = \left( {\frac{t}{{2w}}} \right)^2.$$

Hence, the film thickness *t* must be at least twice the beam width *w* to deter out-of-plane buckling. Therefore, we choose *t* *=* 2.5 μm to guarantee in-plane motion for an aspect ratio of 2.5:1. Then, $\sigma _{\mathrm{c}}^{{\mathrm{op}}} \approx 1.6 \times \sigma _{\mathrm{c}}^{{\mathrm{ip}}}$.

The actuation voltage and current to achieve a targeted displacement of the shuttle are more accurately predicted using the coupled model than with the zeroth-order model. The property values used are listed in Supplementary Table S1. The Ta-to-PS actuation voltage ratio is found to be 1:16 in comparison with 1:14. This is mainly because the real CTE ratio, *α*_Ta_:*α*_PS_, is more than 2:1 (*α*_Ta_ = 6.3 µε/°C^25^ and *α*_PS_ = 2.5 µε/°C^13^ at room temperature). The actuation voltage ratio is found to be independent of displacement. The 16× lower voltage of Ta TAs than of PS TAs means that these actuators can operate at 0.3 V for a measured displacement of 1.5 μm, as shown in subsection ‘Thermal actuator characterization’, which makes them compatible with CMOS (≤1.8 V). Additionally, the power consumption is decreased by 60%, in comparison with the zeroth-order model decrease of 50%. These results are shown in Fig. 1b, where the actuation voltages and power consumptions of PS and Ta TAs at different maximum displacements are presented, and the predicted data previously discussed are circled.

The temperature change due to Joule heating is not uniform along the TA legs. While the anchors are at room temperature, the maximum temperature generally occurs at a location between the anchors and the shuttle. A high operating temperature can lead to softening, plastic deformation, or creep of the structural material. In air, oxidation can affect the maximum temperature, as discussed in subsection “Thermal actuator characterization”. Therefore, a smaller increase in temperature while maintaining the desired displacement/force output is important for achieving reliable TAs. Due to its higher CTE, Ta actuators experience less than half of the maximum temperature change compared to PS. For a measured displacement of 1.5 μm, Ta TAs operate at a temperature change of ~75 °C. This is much lower than *T*_m_/3, above which creep is generally assumed to initiate for metals. The maximum temperature change along the actuator legs is presented in Fig. 1c (olive lines), and the predicted data previously discussed are circled.

Stress is another factor that can plastically deform the structural material or accelerate creep at elevated temperatures. The predicted maximum operating stress near the anchors is also plotted in Fig. 1c (black lines). Because of a slightly higher Young’s modulus, Ta TAs have a higher operating stress than PS. If the displacement is larger than 3 μm, the stress exceeds the yield strength of bulk Ta (~250 MPa)^28^. This issue is addressed in subsection “Preliminary experiments to assess whether Ta can be integrated into MEMS”.

#### Self-actuation

Self-actuation is achieved by raising the ambient temperature, leading to a *uniform* temperature in the actuator legs. An FEA model is developed using ANSYS to predict the displacement of the shuttle as the temperature changes. Only one actuator leg pair is considered, as the displacement does not depend on the number of legs. Boundary conditions include fixed constraints at two ends of the leg pair (anchors) and a temperature change. An example model result of a deformed leg pair subjected to a temperature change, ∆*T* *=* 100 °C, is shown in Supplementary Fig. S1.

The displacement of the shuttle, *δ*, due to a homogeneous temperature change, ∆*T*, can also be obtained analytically, as follows^29^

4 $$\delta = \alpha {\Delta}TL\frac{{{\mathrm{sin}}\left( \theta \right)}}{{\left( {{\mathrm{sin}}^2\left( \theta \right) + \cos ^2\left( \theta \right)\frac{{12I}}{{AL^2}}} \right)}},$$

where *α* is the effective CTE, and *I* = *tw*^3^/12 is the moment of inertia of the actuator leg cross-section.

#### Model summary

The more detailed models support our expectation from the zeroth-order model that Ta has important advantages over PS as a TA structural material because Ta exhibits the following:

(i) a 16× lower driving voltage, compatible with CMOSs,
(ii) a 60% lower electric power consumption,
(iii) a 50% lower operating temperature for the same displacement, and
(iv) the capability of self-actuation.

In the next section, we assess whether Ta is a viable structural material for surface micromachining.

### Preliminary experiments to assess whether Ta can be integrated into MEMS

A thorough investigation of whether Ta can be integrated into a conventional MEMS fabrication process flow as a structural material has not previously been reported. Additionally, the electrothermomechanical model uses the bulk Ta material properties listed in Supplementary Table S1. It remains unclear whether Ta thin films possess comparable properties. To demonstrate Ta as a viable structural material, the following questions must be explicitly answered:

A. Unlike bulk Ta, which assumes the equilibrium bcc *α-*phase, thin film Ta deposits more readily in the metastable tetragonal *β-*phase^30^. Which of these is the better candidate?
B. Bulk Ta has a low yield strength (~250 MPa)^28^, but thin film Ta is generally nanocrystalline. Is it strong enough to survive the bending stress, as predicted in Fig. 1c?
C. PS is typically annealed at 1000 °C to reduce stress^17^. Is it possible to control the Ta film residual stress while eliminating subsequent annealing steps or minimizing their temperatures?
D. Does the Ta thin film possess comparable CTE to bulk Ta?
E. The film thickness is 2.5 μm to prevent out-of-plane buckling. However, if *α-*Ta is chosen, the thickest *α-*Ta feature patterned by conventional reactive ion etching (RIE) with good sidewall protection reported in the literature is only 100 nm^31^. Is it possible to etch a much thicker *α-*Ta film and achieve a vertical sidewall for slender (1 μm wide) TA legs?

This section describes experiments that examine these issues.

#### Phase selection

**Fig. 2:**
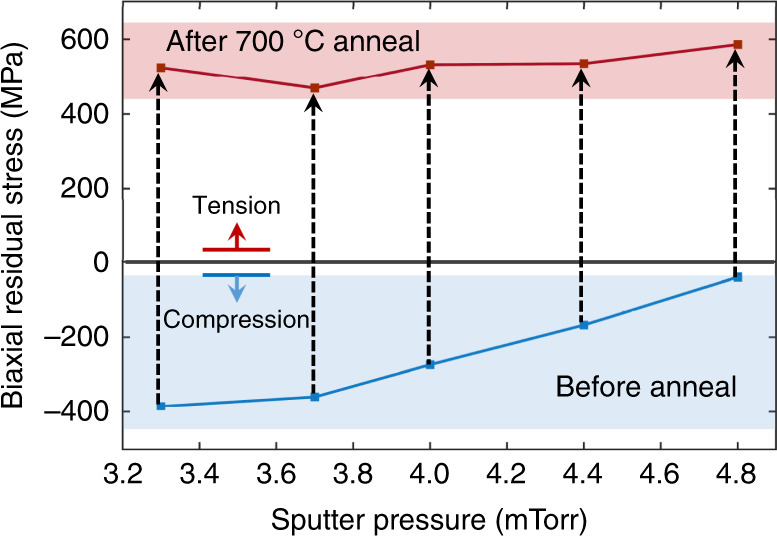
*β*-Ta films exhibit a high tensile stress after anneal. Residual stress of *β*-Ta films as a function of sputter pressure before and after annealing at 700 °C

When deposited directly onto thermally grown oxide, Ta assumes the tetragonal *β*-phase, as indicated by a relatively high resistivity of *ρ* = 173 µΩ cm. This high *ρ* phase (170–210 µΩ cm) is of interest in thin film resistors and heaters and is a potential material for magnetoresistive random access memory technologies. As the sputter pressure decreases, energetic particle bombardment is intensified and results in a higher compressive residual stress^32^. For repeatable operation, a lack of intrinsic (or growth) stress change is needed at the potential operating temperatures. However, the compressive stress in *β*-Ta is fully relaxed, and a tensile biaxial residual stress, $\sigma _{\mathrm{R}}^{\mathrm{b}}$, of 400–600 MPa results after a 700 °C annealing step. This annealing step is performed in an argon-purged rapid thermal anneal (RTA) chamber at atmospheric pressure. To minimize oxidation, the Ta is covered by plasma-enhanced chemical vapor deposition (PECVD) SiO_2_ that is 1 μm thick, which is stripped by RIE after annealing. Figure 2 presents the $\sigma _{\mathrm{R}}^{\mathrm{b}}$ values of blanket *β*-Ta films deposited from 3.3 to 4.8 mTorr before and after annealing.

**Fig. 3:**
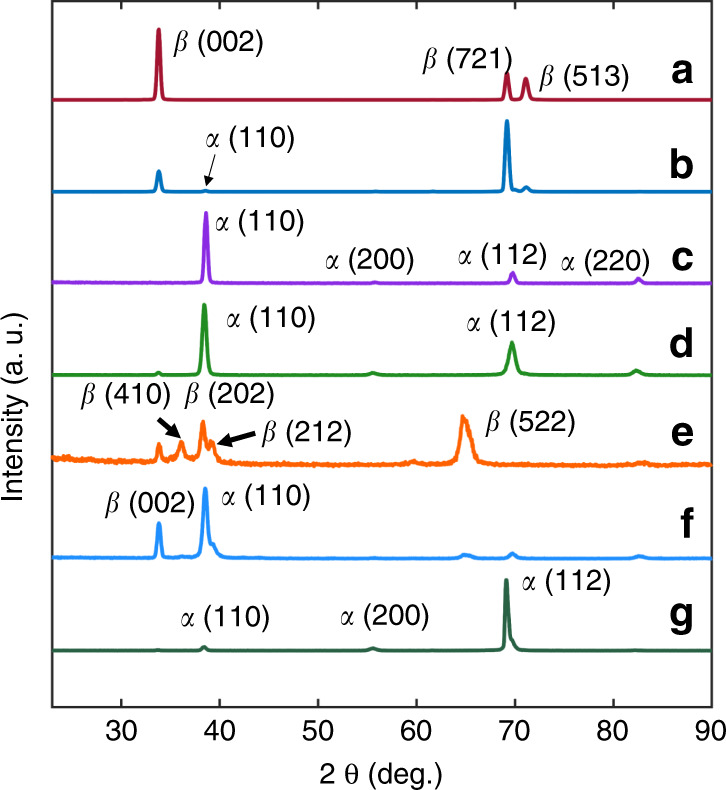
Phase and preferential orientation changes of Ta films under different deposition and annealing conditions. XRD diagrams of **a** as-deposited *β*-Ta films, **b** *β*-Ta films after annealing at 500°C for 20min reveals nucleation of *α*-Ta films, **c** *β*-Ta films after annealing at 700°C for 10min, **d** as-deposited *α*-Ta films on a Cr seed layer, vacuum NOT broken, **e** as-deposited *α*-Ta films on a Cr seed layer, vacuum broken, NO sputter clean, **f** as-deposited *α*-Ta films on a Cr seed layer, sputter clean, and **g** *α*-Ta after hydrogen degas (described in subsection “Full fabrication sequence”)

The $\sigma _{\mathrm{R}}^{\mathrm{b}}$ change results from a *β*-to-*α* phase transformation. The X-ray diffraction (XRD) diagram in Fig. 3a confirms that the as-deposited films are the pure tetragonal *β*-phase. Upon 20 min of annealing at 500 °C, *β*-Ta partially transforms to *α*-Ta, as presented in Fig. 3b. When the temperature is increased to 700 °C, the *β*-to-*α* phase transformation is complete within 10 min, as shown in Fig. 3c. This phase transformation causes the film to contract due to the differences in *β*- and *α*-phase densities and due to significant grain growth^30^. As the contraction is constrained, high tension results^30,33^.

To avoid the phase transformation, the more stable bcc *α*-Ta phase is preferred. This phase exhibits a low *ρ* of 15–60 µΩ cm, making it an attractive choice for lower power TAs. The *α*-phase can grow on a 10-nm-thick Cr seed layer. Its bcc unit cell structure is confirmed by the XRD diagram in Fig. 3d, and *ρ* is measured as 21.4 µΩ cm. However, we find that a freshly prepared seed layer is essential to grow *α*-Ta. If the vacuum is broken between the Cr and Ta depositions, then Ta grows in the pure *β*-phase (Fig. 3e). This is likely due to the immediate oxidation of Cr when it is exposed to air. By sputter cleaning the substrate with a bias voltage of −40 V for 45 s after the vacuum is broken, a mixture of *α*- and *β*-phases with *ρ* *=* 30.0 µΩ cm is obtained, with the most intense peak from *α*-Ta (Fig. 3f). It is likely that sputter cleaning removes most chromium oxide, but residue remains. Therefore, successive Cr/Ta depositions or a sufficiently long sputter-cleaning step is needed to avoid the formation of *β*-Ta.

#### Hardness enhancement

**Fig. 4:**
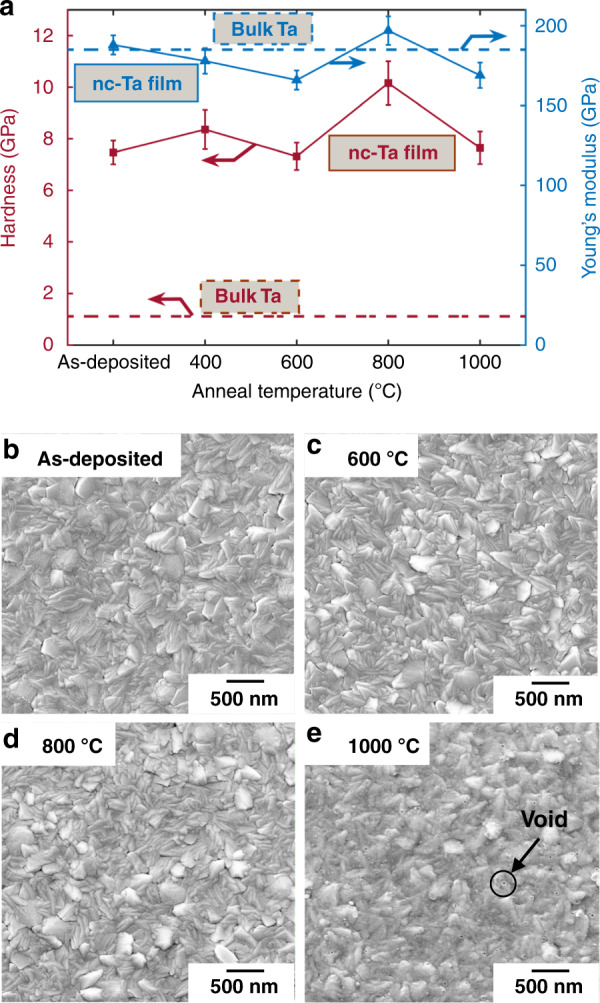
Strength of thin film *α*-Ta remains stable after anneals up to 1000 °C. **a***ɑ*—Hardness and modulus of *α*-Ta films in the as-deposited condition and after annealing at 400–1000°C (solid) and hardness of bulk Ta at room temperature (dashed). *α*-Ta films of 2.5 µm thickness with grain size of about 160 nm in the **b** as-deposited condition and after 30min annealing at **c** 600°C, **d** 800°C, and **e** 1000 °C

We find that the strength of *α*-Ta films is significantly enhanced over that of bulk Ta. The nanoindentation hardness, *H*, is 7.47 ± 0.46 GPa at RT. This corresponds to a yield strength *σ*_y_ ≈ *H*/3 = 2.5 GPa, which is ten times greater than bulk Ta. Therefore, even at a deflection of 4 µm, the maximum bending stress of ~600 MPa, as shown in Fig. 1c, remains well below *σ*_y_ with a safety factor of ~4. A Young’s modulus of 188 ± 6 GPa is also measured at RT, comparable to that of bulk Ta, with a value of 185 GPa^34^. We also measure the hardness and Young’s modulus of *α*-Ta films ex situ after annealing at atmospheric pressure from 400–1000 °C in an argon-purged RTA chamber. These properties are stable even after annealing at 1000 °C. *H* measurements, along with the bulk Ta data at room temperature (RT) from the literature^34^, are shown in Fig. 4a.

This strength enhancement arises from the fine-grained microstructure. Top views of Ta films in the as-deposited condition and after annealing are shown in Fig. 4b–e. According to the Hall-Petch relationship, nanocrystalline Ta should have a higher *σ*_y_ than bulk Ta, which can have a grain size on the order of several hundred micrometers. Guisbiers et al.^35^ obtained a Hall-Petch coefficient of 58 GPa nm^1/2^ by measuring the hardness of Ta films with different grain sizes. Based on this strengthening coefficient, our measured hardness of 7.47 GPa for the as-deposited films corresponds to an average grain size of ~160 nm, which is in reasonable agreement with Fig. 4b. Although no obvious change in grain size is observed after annealing at 600 °C and 800 °C (Fig. 4c, d), voids are formed after annealing at 1000 °C (Fig. 4e).

The lack of noticeable grain growth may be rationalized by the low homologous temperature (i.e., at *T* = 800 °C, *T*/*T*_m_ = 0.33), the relatively short 30 min annealing time, and the inferred grain size of 160 nm. In comparison, Chookajorn et al.^36^ annealed pure W with a 20-nm grain size at *T*/*T*_m_ = 0.37 for 1 week, and grains grew to ~600 nm. The larger initial grain size in Ta and the shorter annealing time indicate that the lack of grain growth inferred here is not unreasonable. Additionally, an increased electrical resistivity of 22.5 µΩ cm and 27.0 µΩ cm (compared to 21.4 µΩ cm) is measured after annealing at 800 °C and 1000 °C, respectively. Since Ta resistivity increases with oxygen content^37^, trace oxygen may be incorporated into the film during the annealing steps, leading to pinning of grain boundaries^38^. Peak broadening could not be detected in the X-ray analysis due to the relatively large grain size. However, the annealing steps do result in a change in the preferential orientation. Compared with the XRD diagram of the as-deposited films (Fig. 3d), the (112) orientation becomes increasingly dominant as the annealing temperature increases, as shown in Fig. S2. The films remain in the *α-*phase, and no new phase is formed. A more detailed microstructural study after annealing is an important topic for future work. Additionally, it is known that fatigue causes grain growth in nanocrystalline metals^39^, but the grain size of Ta TAs undergoing thousands of actuation cycles is not evaluated in this work.

#### Residual stress control

As mentioned above, postdeposition annealing is usually needed to reduce PS residual stress. However, the biaxial residual stress $\sigma _{\mathrm{R}}^{\mathrm{b}}$ of sputter-deposited *α-*Ta can be well controlled during deposition by adjusting the sputter pressure. A compressive $\sigma _{\mathrm{R}}^{\mathrm{b}}$ is observed at lower sputter pressures due to enhanced energetic particle bombardment^32^. This value increases linearly with pressure and becomes tensile at a critical sputter pressure of ≈ 4.8 mTorr. The $\sigma _{\mathrm{R}}^{\mathrm{b}}$ versus sputter pressure slope is shallower than the steep stress change for Ta around the critical sputter pressure reported by Thornton et al.^40^. This makes it easier to achieve near-zero stress, allowing for a greater process margin. The $\sigma _{\mathrm{R}}^{\mathrm{b}}$ versus sputter pressure data of *α-*Ta are presented in Supplementary Fig. S3.

#### Coefficient of thermal expansion

The CTE is a key property needed to predict TA behavior. The stress changes in blanket *α-*Ta films as a function of temperature during heating and cooling cycles in air are shown in Supplementary Fig. S4. For cubic materials, CTE is isotropic. In each cycle, the film deforms thermoelastically, and the slopes are constant and nearly identical from RT to 150 °C. The curve fitting results using the least-squares method are shown in Supplementary Fig. S4. Based on Eq. (5) (see Materials and Methods), the average ∆*α* of the heating and cooling stages is 3.2 ε/°C. Using an *α*_Si_ of 2.7 µε/°C^15^, *α*_Ta_ is determined to be 5.9 µε/°C. This is in good agreement with the published bulk Ta CTE of 6.3 µε/°C^25^. The material properties of bulk Ta, Ta thin films, and PS at room temperature are summarized in Table S2.

#### Reactive ion etching

A major challenge arising from the large aspect ratio of 2.5:1 of the TA legs is vertical etching of the sidewalls. This large thickness-to-width ratio may be associated with severe lateral etching. This leads to a loss of shape and leg width, which can significantly change the output force of TAs^13^. It is known that *α-*Ta is more difficult to pattern using RIE than *β*-Ta^30,41^. Much work has established that due to the high volatility of Ta fluoride and chloride, Ta can be etched by conventional plasma etching based on fluorine^42–44^, chlorine^45^, or interhalogen compounds^44^. However, these studies were mostly carried out for *β*-Ta or unpatterned *α*-Ta films. To the best of our knowledge, the thickest reported *α-*Ta feature patterned by conventional RIE with good sidewall protection is 100 nm^31^. This was achieved using a SiCl_4_–NF_3_ gas mixture that is not typically available in a standard cleanroom environment. Thicker *α-*Ta structures of 350–400 nm were also etched, but they were patterned by an unconventional electron cyclotron resonance ion stream with a mixture of chlorine and fluorine gases^46^.

**Fig. 5:**
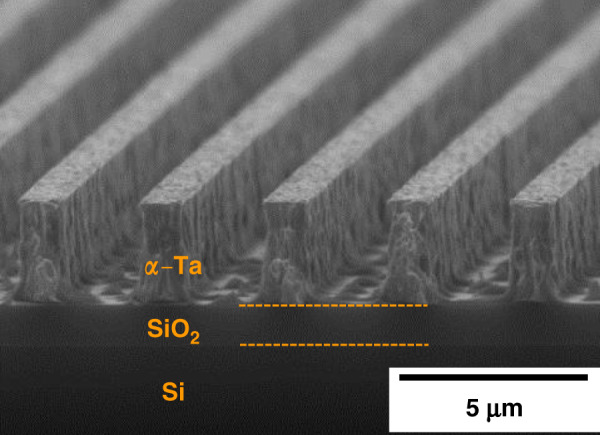
A high thickness-to-width ratio of 2.5:1 is achieved with minimal lateral etch. *α*-Ta RIE showing vertical sidewalls. Remaining islands in the field areas are swept away upon release

Using fluorine-based RIE, we perform work with an extensive scope to improve sidewall protection for the 1 μm feature. We find that O_2_, which is often added to etch chemicals to release more fluorine and to boost the etching rate^42,43,45^, can substantially enhance lateral etching. In contrast, the addition of Ar improves anisotropy. We also find that *α-*Ta exhibits a significant macroloading effect: sidewall protection is generally much better for a wafer than for a chip. Other process parameters, including pressure, gas flow and power, are also found to influence the extent of the lateral etching. Our efforts resulted in minimal lateral etching and a vertical sidewall profile after a 1 h RIE step (Fig. 5) under the optimal conditions.

### Full fabrication sequence

The evidence from subsection “Preliminary experiments to assess whether Ta can be integrated into MEMS” indicates that thin film *α-*Ta possesses the properties required for a promising TA material and, importantly, that it can be processed by conventional MEMS fabrication methods. Therefore, in this section, we evaluate a full fabrication sequence for Ta TAs. Outstanding issues are as follows:

1. Although the legs in TAs are fixed on both sides, the shuttle itself will bend if the residual stress gradient is too high. Can the stress gradient be sufficiently well controlled?
2. Ta is reported to survive a buffered hydrofluoric acid (BHF) release etching step. However, hydrogen can readily diffuse into metal films and change the residual stress. Does this occur, and can it be reversed?

**Fig. 6:**
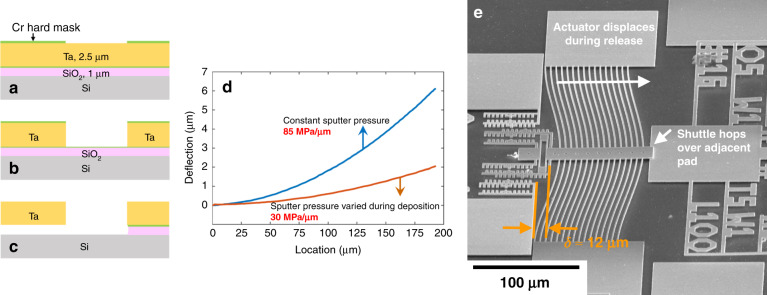
*α*-Ta films are integrated into a full MEMS fabrication process but issues of large stress gradient and hydrogen incorporation arise. Fabrication process: **a** 2.5-μm-thick α-Ta is deposited on the substrate, followed by deposition of a 45-nm-thick Cr hard mask which is patterned by photolithography and ion mill, **b** Ta is RIE’d using fluorine-based chemicals, and **c** the Cr seed layer and hard mask and the sacrificial oxide are stripped, structures are rendered freestanding by CPD and degassed at 500°C in UHV to remove injected hydrogen in the release step, **d** deflections of 193-μm-long cantilever beams, and **e** a 12-µm displacement of a TA after BHF release due to hydrogen injection, corresponding to 1.24 GPa compressive stress

The proposed full fabrication process flow is shown in Fig. 6a–c. Following deposition of a 2.5-μm-thick *α*-Ta film, a 45-nm-thick Cr layer is deposited as an etch hard mask. Because the hard mask is thin, a positive photoresist with only a 400 nm thickness can be used to maximize the resolution. The Cr hard mask is ion milled, as assisted by end-point detection to avoid any redeposition effect, and the photoresist is stripped by a gentle oxygen plasma application (Fig. 6a). Next, the Ta film is subjected to RIE using CF_4_ and Ar (Fig. 6b). The etch rate is ~42 nm/min. Subsequently, the Cr hard mask is stripped selectively by a Cr wet etchant, followed by the removal of the sacrificial thermal oxide at room temperature by BHF (5 parts 40% NH4F:1 part 49% HF) for 1 h. Critical point drying (CPD) results in the freestanding structures (Fig. 6c).

At a constant sputter pressure, the stress gradient is 85 MPa/μm (Fig. 6d), which causes unacceptable curvature in the shuttle, as it contacts the substrate, as shown in Supplementary Fig. S5. Therefore, during the Ta deposition step, the sputter pressure is varied to balance the intrinsic stress gradient. Supplementary Table S3 shows a protocol in which the sputter pressure is gradually decreased. The stress gradient can be determined from the tip deflection of cantilever beams, *δ*, using the following equation^47^:

6 $$\frac{{{\mathrm{d}}\sigma _{\mathrm{R}}^{\mathrm{u}}}}{{{\mathrm{d}}z}} = \frac{E}{{1 - \nu }}\frac{2}{{l^2}}\delta,$$

where *l* is the beam length, $\sigma _{\mathrm{R}}^{\mathrm{u}}$ is the uniaxial residual stress, and *z* is the out-of-plane direction. The tip deflection is measured by interferometry. The cantilever beam bends up, meaning that the stress shifts toward tension as the film deposition continues. The results for “sputter pressure varied during deposition” are presented in Fig. 6d. By utilizing decreasing sputter pressures, the stress gradient decreases to 30 MPa/μm, and the tip deflection is almost three times smaller. As sputter pressures both higher and lower than the critical sputter pressure are used, the average $\sigma _{\mathrm{R}}^{\mathrm{b}}$ of the as-deposited blanket Ta film remains at a low level of approximately −40 MPa. With this sequence, the released TA shuttle becomes freestanding. The stress gradient is measured after a hydrogen degas annealing step, as explained next.

After the BHF release step, we observe that the residual stress shifts substantially toward compression. The residual stress changes increase linearly with BHF exposure time and reach −1 GPa after 150 min. This is because during the release step, BHF injects atomic hydrogen into the film, which expands the crystal structure and results in compressive stress, as reported in the literature^48^. To remove hydrogen and recover the as-deposited residual stress, we degassed the sample at 500 °C under ultrahigh vacuum (UHV). This annealing step leads to a change in the preferential orientation of the film (Fig. 3g), similar to the data in Fig. S2. After degassing the sample, the stress, extracted using fixed-fixed beams, is largely recovered but is still 50–60 MPa more compressive than the initial stress. More details about stress recovery can be found in the literature^48^.

**Fig. 7:**
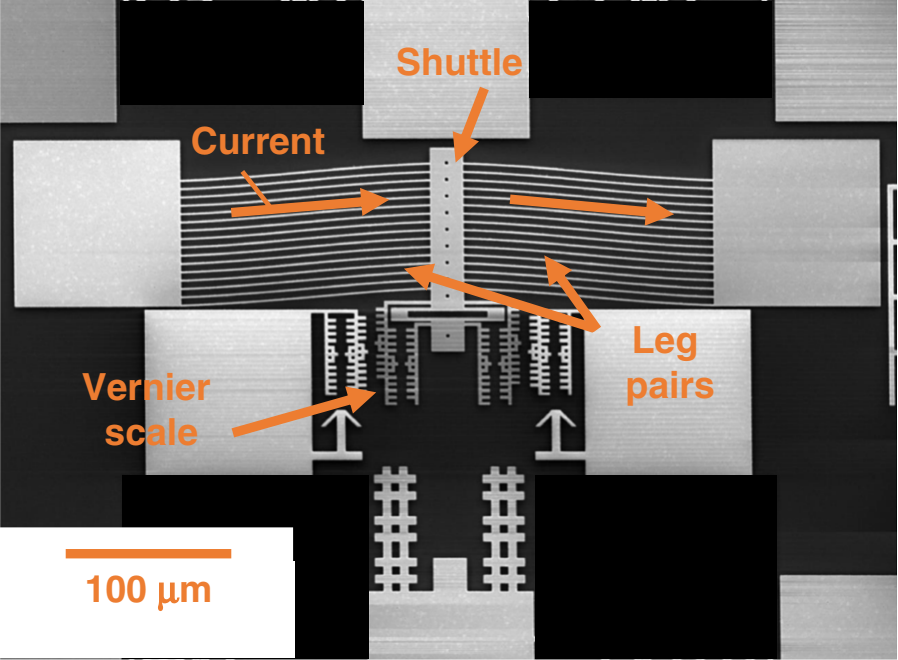
Functional *α*-Ta TAs are fabricated after stress gradient reduction and hydrogen degas. Top view of freestanding Ta thermal actuator. In-plane deflection *δ* ≈ 5µm after hydrogen degas step

Following the process flow in Fig. 6a–c, *α-*Ta TAs are successfully fabricated. The TA shown in Fig. 7 has 16 leg pairs, with the same leg geometry as in Fig. 1. TA legs with this geometry are used for characterization, as discussed next.

### Thermal actuator characterization

#### As-released structure

Enormous and unexpected in-plane TA displacements of ~12 µm are initially observed after release (Fig. 6e). This corresponds to a residual compressive stress of 1.24 GPa, and this problem lead us to discover the hydrogen injection phenomenon discussed above and solve it using the postrelease UHV annealing step^48^. With this annealing step, the in-plane displacement after release is ~5 µm, corresponding to a stress of −340 MPa. The TA is fabricated using a blanket Ta film with a uniaxial stress of approximately −27 MPa. Therefore, the stress change after the UHV annealing step is approximately −310 MPa, which is higher than that measured in fixed-fixed beams (−60 MPa)^48^. The reason for this result is likely that TA legs are much narrower (1 µm wide) than fixed-fixed beams (10 or 20 μm wide) and have a higher surface-to-volume ratio. This makes them absorb more hydrogen than the wide fixed-fixed beams, meaning that more hydrogen may remain in the film after the UHV step. We deem this adequate for TA characterization, but in future work, a final stress near zero is, in principle, achievable by further adjustments of the sputter pressure or by a longer degassing period. We will present other approaches that resolve this problem in a future publication.

Figure 5 shows that the Ta RIE leaves residue between the lines. We find that these small islands are swept away during the release process, and therefore, the appearance of the chips is clean, as seen in Fig. 7.

#### Actuation by Joule heating

All displacements reported below are in-plane and are with respect to the displacement after release. The displacements are measured as a function of current in air and compared to the electrothermomechanical model results. A Vernier scale is imaged under a 50× objective lens during actuation and is used to determine the displacement of the shuttle by pattern matching, which has a resolution of ~5 nm. Ta is susceptible to internal oxidation^49^, and we observe oxidation at 100 °C without protection. The measurements reported are performed with TAs protected by a 20-nm-thick Al_2_O_3_ passivation layer deposited by atomic layer deposition (ALD), which increases the oxidation-resistant temperature to ~150 °C. While further improved passivation is a topic for future research, under high vacuum conditions that can be achieved in test chambers^50^ or in electron microscopes, the operating temperature is likely to be substantially higher (minimal oxidation up to 750 °C is reported in the literature^51^).

**Fig. 8:**
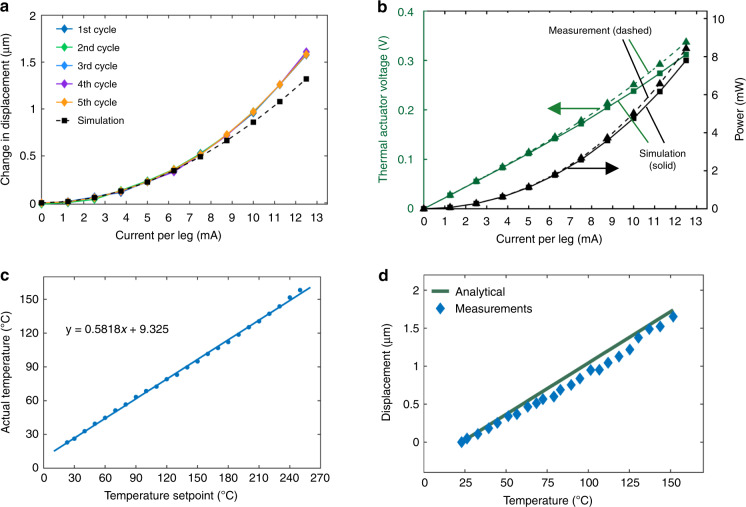
Characterization of *α*-Ta TAs by Joule heating and self-actuation is in good agreement with simulations. **a** Displacement as a function of current per leg for five actuation cycles, **b** modeled and measured actuation voltage and electric power consumption as a function of current per leg, **c** temperature calibration. The abscissa is the hotplate temperature setpoint and the ordinate is the TA actual temperature and **d** self-actuation displacement as a function of temperature

The displacement of a TA as a function of current per leg for five actuation cycles is plotted in Fig. 8a (solid line, diamond markers), along with the model results (dashed line, square markers). Repeatable actuation for five actuation cycles is demonstrated. The measurements show agreement with the model up to 6.3 mA/leg, but displacement is enhanced at higher current levels. We attribute this result to a higher actual leg temperature than the modeled temperature, as explained next. The model considers widely spaced leg pairs and assumes that they are thermally isolated from each other, as is valid when the separation is ~100 μm or more^13^. However, in our design, the heated leg pairs are spaced by only 10 μm. This leads to less efficient heat dissipation and an overall higher leg temperature for a given current. The effect becomes increasingly important as heat generation increases at higher currents. This is verified by our observation that TAs with the same geometry but with a leg spacing of only 5 μm in fact generate even greater displacement at the same current due to this self-heating effect.

Additionally, the actuation voltage and electric power consumption are measured as a function of current per leg. They are also predicted by the electrothermomechanical model, as described in subsection “Model”, using the measured Ta thin film properties. The as-fabricated TAs have 16 leg pairs, but only two leg pairs are modeled, so the measured power consumption is divided by 8 to normalize this difference. These results are plotted in Fig. 8b. An overall agreement between the measurements and the model is evident. The measured actuation voltage and power are slightly higher at high currents due to the self-heating effect, as previously explained, which slightly increases the electrical resistivity. The low power consumption and actuation voltage (below 0.35 V) of Ta TAs are confirmed by these measurements.

The repeatable results indicate that Ta oxidation is a minor issue up to the estimated maximum leg temperature (including self-heating) of 120 °C. Including the residual stress inferred from the 0 V displacement, our model calculates that the maximum operating stress in the legs is approximately +500 MPa (tension) and −610 MPa (compression). Similarly, the strength of nanocrystalline Ta is well above the operating stress.

#### Self-actuation

Self-actuation is tested in air on a hot plate using optical microscopy, and displacements are again measured using a Vernier scale. Temperature calibration is necessary because heat transfer is imperfect. Therefore, we measure the resistivity of actuator legs in situ using the four-point probe configuration. By knowing the temperature coefficient of resistance (TCR) of Ta, +3800 p.p.m./°C^52^, the actual temperature of the TA legs, *T*_act_, is deduced after waiting 15 min to establish a steady-state condition at each setpoint temperature, *T*_sp_. Above the reference temperature at RT, *T*_act_ is ~60% of *T*_sp_, revealing the critical importance of calibration. The *T*_act_ values are plotted in Fig. 8c.

An effective CTE of Δ*α* = 3.2 µε/°C, i.e., the difference between the CTE*s* of Ta and the Si substrate, is used for the models. The FEM model results are essentially identical to the analytical model results. For the measurements, the temperature is stepped up in increments of 10 °C. Over the range from RT to 150 °C, the agreement with the model is good. The measured self-actuation in-plane displacement as a function of *T*_act_ and that of the analytical model are shown in Fig. 8d.

For this specific TA design, the temperature sensitivity is ~14 nm/°C. This can be adjusted by changing the TA leg geometry and can be further improved by certain amplification designs, such as cascaded structures^12^, for different applications. In addition, a comprehensive comparison of V-shaped TAs made of Ta, PS, Ni, and SU-8 can be found in Tables S4. A, B.

## Conclusions

This work demonstrates the promising application of Ta as a new structural material for TAs with advantages over polysilicon including low voltage and lower operating and process temperatures. The demonstration is based on systematic studies of material properties, including Young’s modulus, hardness, and CTE; key fabrication challenges, including phase changes, anisotropic etching, residual stress control, hydrogen incorporation, and desorption; and modeling and measurements of two different actuation mechanisms, i.e., conventional actuation by Joule heating and self-actuation. The following conclusions are supported by the evidence provided:

- The Ta to polysilicon actuation voltage ratio is approximately 1:16, making Ta TAs compatible with CMOS. The electric power consumption of Ta TAs is decreased by 60% for the same displacement (Fig. 1b).
- Ta TAs can operate at half the maximum temperature change of polysilicon for the same displacement. Although the leg bending stress is slightly higher, Ta TAs retain a high safety factor (Fig. 1c).
- A *β*-to-*α* phase transformation leads to full relaxation of the compressive stress and a tensile stress of 400–600 MPa. It is preferred to deposit Ta in the bcc *α-*phase by introducing a Cr seed layer (Figs. 2 and 3).
- *α*-Ta films have a significantly larger hardness value of 7.47 ± 0.46 GPa at RT than bulk Ta. This corresponds to a ≈10× higher yield strength. The hardness and Young’s modulus remain stable even after annealing up to 1000 °C (Fig. 4a).
- The average biaxial residual stress of *α-*Ta films can be well controlled by adjusting the sputter pressure, which is characterized by a gradual stress change near the critical sputter pressure (Supplementary Fig. S3).
- The CTE of *α-*Ta films is ~5.9 ε/°C, comparable to that of bulk Ta (Supplementary Fig. S4).
- A vertical sidewall profile and minimal lateral etching are achievable for the TA legs (Fig. 5).
- By varying the sputter pressure in situ, the stress gradient is reduced from 85 MPa/μm to 30 MPa/μm (Fig. 6d).
- Following conventional surface micromachining (Fig. 6a–c), *α*-Ta TAs are successfully fabricated (Fig. 7).
- Repeatable actuation by Joule heating is demonstrated. Overall, the measured displacement agrees with the modeling but deviates at a high current due to limited heat dissipation (Fig. 8a).
- Self-actuation is demonstrated by increasing the ambient temperature. The measurements agree well with the modeling and analytical solutions (Fig. 8d).

The maximum TA displacement in air can be increased by developing improved passivation layers. While not explored herein, this micromachined structural Ta material can, in principle, also be used to construct many other MEMS devices, including but not limited to fixed-fixed beams^48^, comb drives, resonators, optical components, accelerometers, and gyroscopes. We note that these devices do not require elevated temperatures to operate. The maximum process temperature is 500 °C. Therefore, Ta is a candidate structural material for post-CMOS processing^53,54^.

## Materials and methods

The substrates used are 4″ (100) Si wafers with a 1-µm-thick thermally grown oxide layer. A load-locked DC magnetron sputtering system (CVC Connexion Cluster Tool) is used to deposit Ta films at a base pressure lower than 7 × 10^−8^ Torr without intentional heating or biasing using Ar as the working gas. At a power of 250 W, the deposition rate is ~19 nm/min. Crystal structures are determined on blanket films using XRD under conventional reflective configurations with a copper K_α_ source operated at 45 kV and 40 mA (Philips X’pert Pro MRD, model no. PW3040/60). Resistivity is measured using a standard four-point probe configuration. The Young’s modulus and hardness are determined by nanoindentation^55^.

Based on the Stoney equation^56^, the biaxial residual stress $\sigma _{\mathrm{R}}^{\mathrm{b}}$ is found by measuring the curvature change in substrates before and after deposition using the laser scanning method with a Tencor Flexus tool (FLX-2320). The CTE of Ta is determined using the same tool and method by measuring the stress change as the temperature varies from RT to 150 °C. The relationship between the biaxial stress change, ${\mathrm{{\Delta}}}\sigma _{\mathrm{R}}^{\mathrm{b}}$, and the temperature change, Δ*T*, is as follows:

5 $${\mathrm{{\Delta}}}\sigma _{\mathrm{R}}^{\mathrm{b}} = \frac{E}{{1 - \nu }}{\Delta}\alpha {\Delta}T,$$

where *E* and *v* are the Young’s modulus and Poisson’s ratio of the films, respectively, and Δ*α* is the difference in CTE values between the Ta film and the Si substrate. The CTE of single crystalline Si is well known. Therefore, the CTE of Ta films can be determined by plotting ${\mathrm{{\Delta}}}\sigma _{\mathrm{R}}^{\mathrm{b}} - {\Delta}T$ and finding the slope.

The optimal conditions for Ta RIE are a CF_4_ flow of 6 sccm, an Ar flow of 30 sccm, a pressure of 20 mTorr, and a power of 44 W.

## Supplementary information

Supplemental information
