# Supplementary material for: Demonstration of tantalum as a structural material for MEMS thermal actuators
Source: Microsyst Nanoeng. 2021 Jan 15;7:6. doi: 10.1038/s41378-020-00232-z (PMC8433199; doi:10.1038/s41378-020-00232-z)
Supplement: Supplementary file 1 — Supplemental information [file 41378_2020_232_MOESM1_ESM.docx]

Supplementary Information

^a^Longchang Ni, ^a,b^Ryan M. Pocratsky and ^a*^Maarten P. de Boer

^a^CMU Mechanical Engineering Dept., 5000 Forbes Ave., Pittsburgh, PA 15213

^b^Current address: Fischione Instruments, 9003 Corporate Cir, Export, PA 15632

^*^Corresponding author. Email: [mpdebo@andrew.cmu.edu](mailto:mpdebo@andrew.cmu.edu)

This document contains supplementary figures and tables as follows.


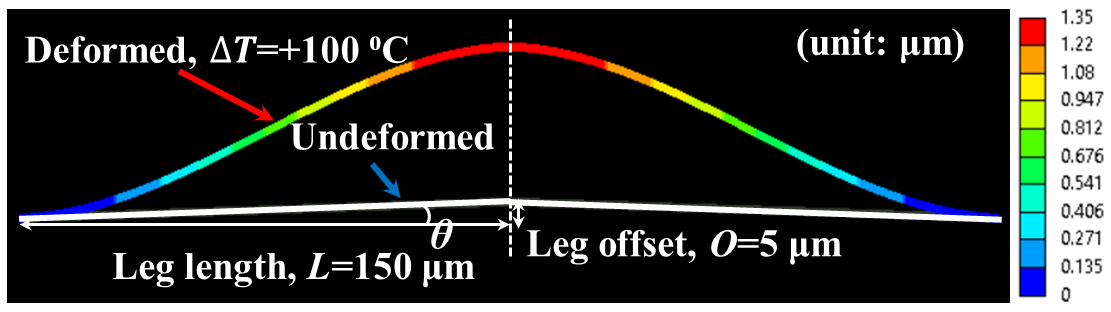


Fig. S1 Thermal actuator leg pair geometry and FEM simulation revealing the displacement of the actuator subject to homogeneous temperature change $\Delta T$=100 °C (displacement exaggerated by 35X to be visible)


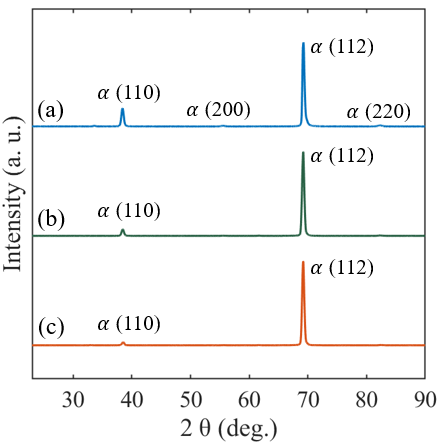


Fig. S2 X-ray diffraction diagrams of *α-*Ta films after anneals at (a) 600 °C, (b) 800 °C and (c) 1000 °C. This information indicates that the texture is evolving more heavily towards (112).


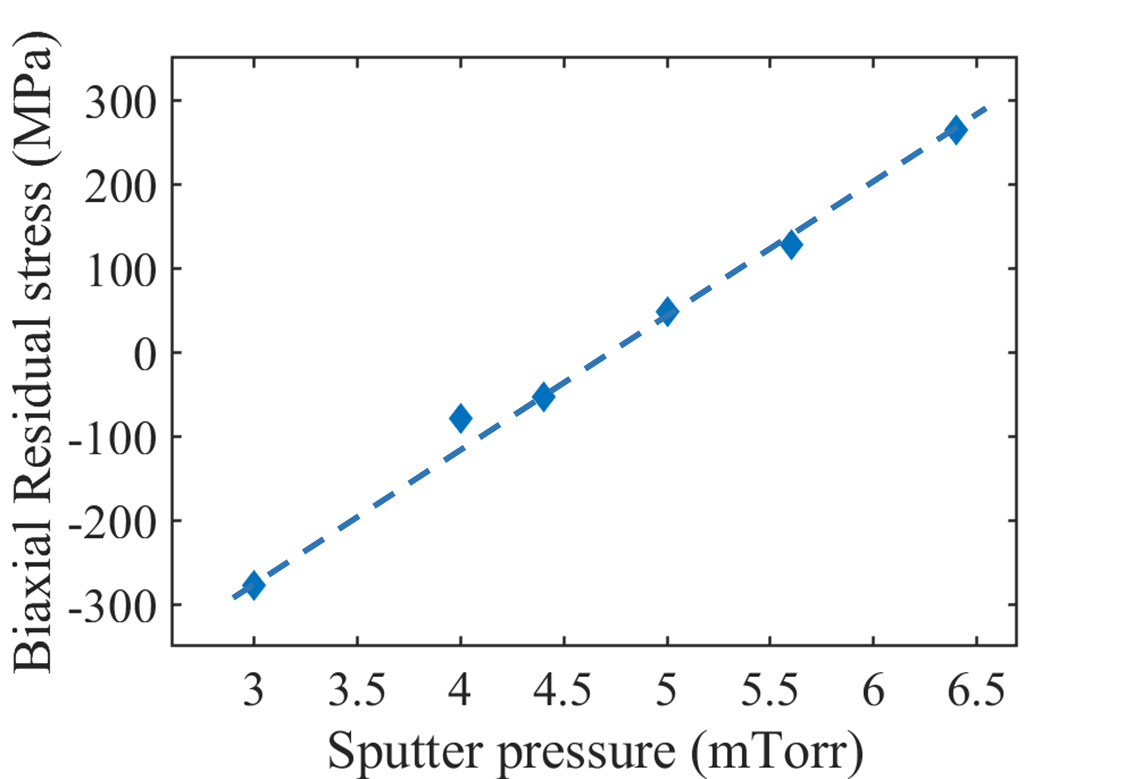


Fig. S3 Residual stress of *α*-Ta films as a function of sputter pressure. A shallow slope is observed, making stress control manageable.


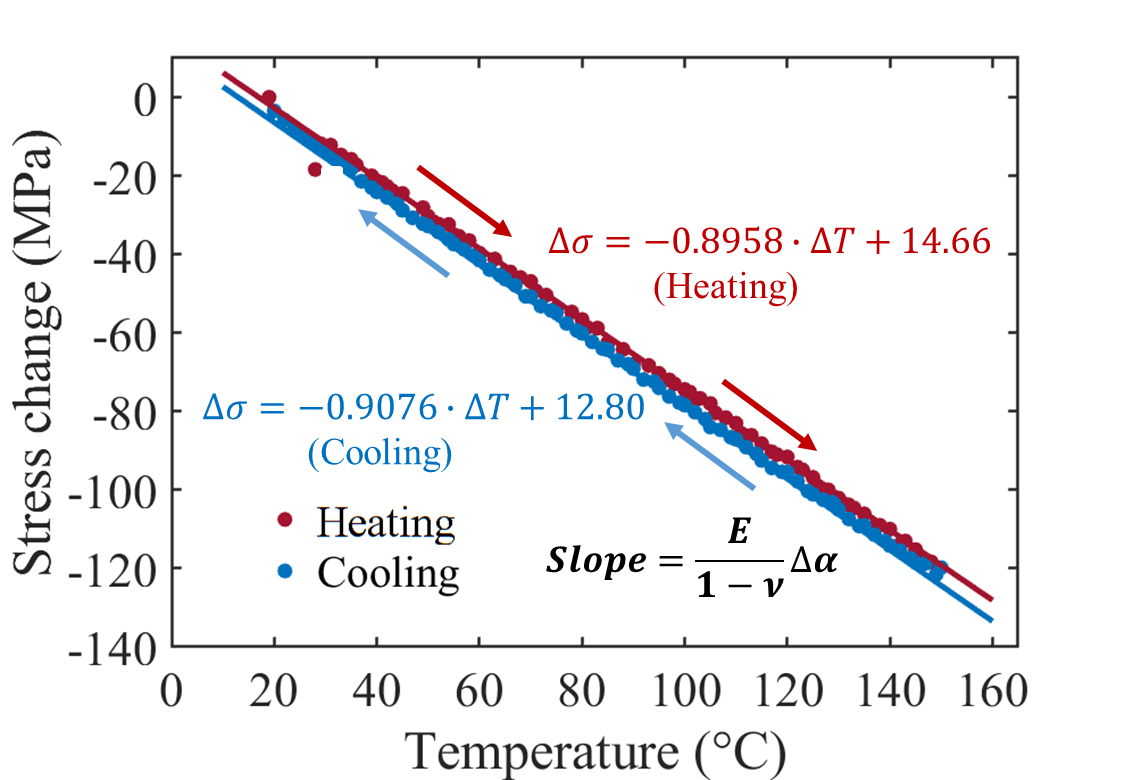


Fig. S4 Residual stress change, measured by wafer curvature, as a function of temperature for *α*-Ta films. Note that absolute temperatures rather than *∆T* are plotted but this does not influence the slopes.


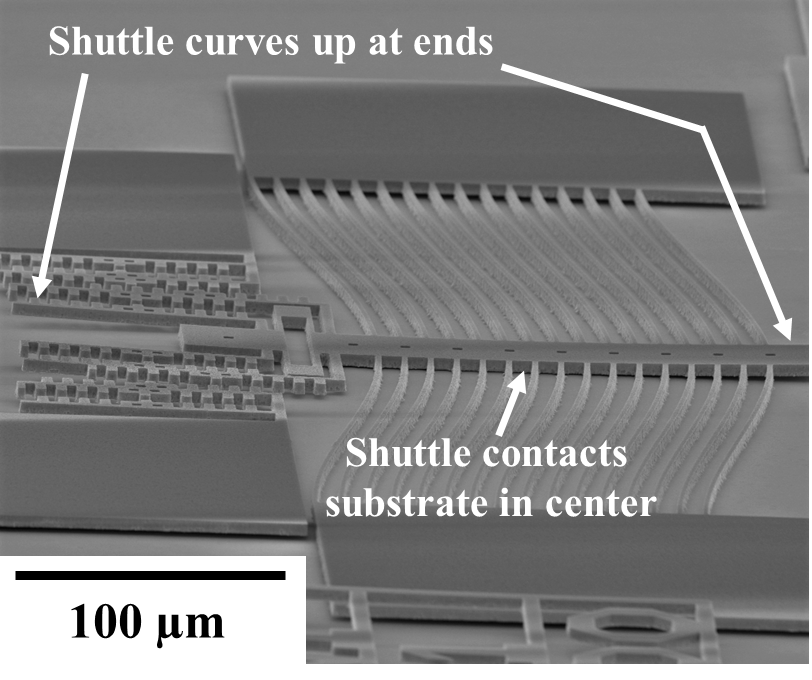


Fig. S5 A TA in which the shuttle contacts the substrate due to large curvature.

Table S1 Material properties for modeling (temperature, *T*, is in °C)

|  | Ta | PS |
| --- | --- | --- |
| Resistivity  (Ω·μm) | 0.157201 $+$ 4.06757×10^-4^*T* $-$ 2.32200×10^-8^*T*^2^ (ref. ^1^) | *1)* (2.9713×10^-2^)*T* $+$ 20.858 (*T*<300 °C)  *2)* (6.1600×10^-5^)*T*^2^ $-$ (7.2473×10^-3^)*T* $+$ 26.402 (300 °C < *T* < 700 °C)  *3)* (8.264×10^-2^)*T*$-$ 8.8551 ($T$>700 °C) (ref. ^2^) |
| Thermal conductivity (W/(m·°C)) | *1)* 57.33 $+$ 0.003968*T* (*T* < 1526.8 °C)  *2)* 64.7 $-$ 0.007131*T* $+$ 5.704×10^-6^*T ^2^* $-$ 1.043×10^-9^*T*^3^ (ref^. 3^) | 1/((-2.2×10^-11^)*T*^3^ $+$ (9.0×10^-8^)*T*^2^-  (1.0×10^-5^)*T* $+$ 0.014) (ref. ^2^) |
| Coefficient of thermal expansion  (1/°C) | 6.41×10^-6^ $+$ 1.1878×10^-^^9^*T*  (ref. ^4^) | (3.725×(1-exp(-5.88×10^-3^(*T - ­*125))) + (5.548×10^-4^)*T*)×10^-6^  (ref. ^2^) |
| Young’s modulus (GPa) | 188 (determined by nanoindentation) | 164 (ref. ^5^) |

Table S2 Material properties of bulk Ta, Ta thin films and PS at room temperature

|  | Bulk Ta | Ta thin films | PS |
| --- | --- | --- | --- |
| Resistivity (μΩ·cm) | 16.8 (ref. ^1^) | 21.4 | 2150 (ref. ^2^) |
| Thermal conductivity (W/(m·°C)) | 57.4 (ref. ^3^) | - | 72 (ref. ^2^) |
| CTE (µ$\varepsilon$/°C) | 6.3 (ref. ^4^) | 5.9 | 2.5 (ref. ^2^) |
| Young’s modulus (GPa) | 185 (ref. ^6^) | 188 | 164 (ref. ^5^) |
| Hardness (GPa) | 1.17 (ref. ^7^) | 7.47 | 9 (ref. ^8^) |
| Yield strength (GPa) | 0.25 (ref. ^7^) | 2.5 ($=H$/3) | 2.69-3.37 (ref. ^9^) |

Table S3 Varying sputter pressures of Ta deposition

| Thickness (μm) | Time (min) | Pressure (mTorr) |
| --- | --- | --- |
| 0 – 0.2 | 0 – 10.6 | 6.5 |
| 0.2 – 0.4 | 10.6 – 21.2 | 5.0 |
| 0.4 – 0.9 | 21.2 – 47.8 | 4.5 |
| 0.9 – 1.2 | 47.8 – 63.7 | 4.4 |
| 1.2 – 2.5 | 63.7 – 132.8 | 4.0 |

Table S4.A Comparison of Joule heating actuated V-shaped MEMS TAs

|  | *α-*Ta | PS ^*^ | PS (ref. ^2^) | SU-8 (ref. ^10^) |
| --- | --- | --- | --- | --- |
| Max. displacement (μm) | 1.6 | 1.6 | 12 | 5.7 |
| Actuation voltage (V) | 0.34 | 4.91 | 7.0 | <0.2 |
| Power consumption (mW) | 8.4 | 15.6 | 100 | - |
| Max Operation temperature (°C) | 119 ^**^ | 207 | 550 | 80 |
| Leg dimensions (*l×w×t*, μm) | 150×1×2.5 | 150×1×2.5 | 300×4×6.4 | $t$*=*45 ($l, w$ unspecified) |
| Leg aspect ratio (*t*/*w*) | 2.5 | 2.5 | 1.6 | - |
| Fabrication process | SMM | SMM | SMM | UV-LIGA |

* Performance predicted by the model at a displacement of 1.6 μm using the same TA geometry as Ta

** Predicted by the model

*** SMM = Surface micromachining

Table S4.B Comparison of self-actuated V-shaped MEMS TAs

|  | *α-*Ta | Ni (ref. ^11^) |
| --- | --- | --- |
| Max. displacement (μm) | 1.6 | 80 |
| Max Operation temperature (°C) | 119 | 100 |
| Leg dimensions (*l×w×t*, μm) | 150×1×2.5 | 361×40×15 |
| Leg aspect ratio (*t*/*w*) | 2.5 | 0.375 |
| Fabrication process | SMM | LIGA |

Tables S4.A and S4.B present comparisons of joule heated and self-actuated TAs, respectively, made of *α-*Ta with other structural materials that have been fabricated and reported in technical literature. This only provides a general comparison as some TA properties also depend on the TA geometries of specific designs. Modeled performance of PS TAs with the same geometry as Ta is also included in Table S4.A. In general, the main advantages of Ta TAs include capability of self-actuation, low actuation voltage compatible with CMOS, reduced dimensions and mechanical stability. Also, a lower electric power is needed for actuation compared with the dominant PS TAs. TAs made of non-conductive SU-8 have an even lower actuation voltage, but this arises from the co-fabricated conductive heater that is usually a low resistivity metal, such as copper. Other than PS, Ta is the only structural material that is compatible with surface micromachining for TA fabrication. This brings about at least 10 times reduction in the overall size. With the highest leg aspect ratio, Ta TAs are most mechanically stable as out-of-plane motion is prevented.

The main drawback of Ta TAs is their relatively low operation temperature, as limited by oxidation. This also limits the achievable displacement. Efforts to develop an oxidation-resistant coating are undergoing. But this can be resolved if TAs are operated in high vacuum. Additionally, the displacement depends on the TA geometry and scales with the leg length. The Ta TAs tested in this work have a relatively small leg length of 150 μm compared with the PS and Ni TAs in the last two columns of Table S4.A. Thus, the displacement can be further increased by changing leg geometries. Also, as mentioned in Subsection IV, certain amplification designs such as cascaded structures can be utilized for larger displacement.

**References**

1. Taylor, R. E., Kimbrough, W. D. & Powell, R. W. Thermophysical properties of tantalum, tungsten, and tantalum-10 wt. per cent tungsten at high temperatures. *J. Less-Common Met.* **24**, 369–382 (1971).

2. Baker, M. S., Plass, R. A., Headley, T. J. & Walraven, J. A. *Final report : compliant thermo-mechanical MEMS actuators, LDRD #52553. Sandia Report No. Sand2004-6635*. (2004). doi:10.2172/920746

3. Touloukian, Y. S. Thermophysical Properties of Matter vol 1. *J. Chem. Inf. Model.* (1973). doi:10.1017/CBO9781107415324.004

4. Touloukian, Y. S. & Ho, C. Y. Thermophysical Properties of Matter: Thermal Expansion of Metallic Elements and Alloys. **12**, (1975).

5. Jensen, B. D., De Boer, M. P., Masters, N. D., Bitsie, F. & LaVan, D. A. Interferometry of actuated microcantilevers to determine material properties and test structure nonidealities in MEMS. *J. Microelectromechanical Syst.* **10**, 336–346 (2001).

6. Hodgson, D., Wu, M. & Biermann, R. ASM Handbook, Volume 2, Properties and Selection, Nonferrous Alloys and Special-Purpose Materials. (1990).

7. Zhang, M., Yang, B., Chu, J. & Nieh, T. G. Hardness enhancement in nanocrystalline tantalum thin films. *Scr. Mater.* **54**, 1227–1230 (2006).

8. Sundararajan, S. & Bhushan, B. Micro/nanotribological studies of poly silicon and SiC films for MEMS applications. *Wear* **217**, 251–261 (1998).

9. Koskinen, J., Steinwall, J. E., Soave, R. & Johnson, H. H. Microtensile testing of free-standing polysilicon fibers of various grain sizes. *J. Micromechanics Microengineering* **3**, 13–17 (1993).

10. Zhang, R., Chu, J., Wang, H. & Chen, Z. A multipurpose electrothermal microgripper for biological micro-manipulation. *Microsyst. Technol.* **19**, 89–97 (2013).

11. Steiner, H., Keplinger, F., Schalko, J., Hortschitz, W. & Stifter, M. Highly Efficient Passive Thermal Micro-Actuator. *J. Microelectromechanical Syst.* **24**, 1981–1988 (2015).
